# Mechanistic insights from the atomic-level quaternary structure of short-lived GPCR oligomers in live cells

**DOI:** 10.21203/rs.3.rs-4683780/v1

**Published:** 2024-07-19

**Authors:** Michael R. Stoneman, Koki Yokoi, Gabriel Biener, Thomas D. Killeen, Dhruba P. Adhikari, Sadia Rahman, Kaleeckal G. Harikumar, Laurence J. Miller, Valerică Raicu

**Affiliations:** 1 Department of Physics, University of Wisconsin-Milwaukee, WI 53211, USA; 2 Department of Molecular Pharmacology and Experimental Therapeutics, Mayo Clinic, Scottsdale, AZ, USA

## Abstract

The functional significance of the interactions between proteins in living cells to form short-lived quaternary structures cannot be overemphasized. Yet, quaternary structure information is not captured by current methods, neither can those methods determine structure within living cells. The dynamic versatility, abundance, and functional diversity of G protein-coupled receptors (GPCRs) pose myriad challenges to existing technologies but also present these proteins as the ideal testbed for new technologies to investigate the complex inter-regulation of receptor-ligand, receptor-receptor, and receptor-downstream effector interfaces in living cells. Here, we present development and use of a novel method capable of overcoming existing challenges by combining distributions (or spectrograms) of FRET efficiencies from populations of fluorescently tagged proteins associating into oligomeric complexes in live cells with diffusion-like trajectories of FRET donors and acceptors obtained from molecular dynamics (MD) simulations. Our approach provides an atom-level picture of the binding interfaces within oligomers of the human secretin receptor (hSecR) in live cells and allows for extraction of mechanistic insights into the function of GPCRs oligomerization. This FRET-MD spectrometry approach is a robust platform for investigating protein-protein binding mechanisms and opens a new avenue for investigating stable as well as fleeting quaternary structures of any membrane proteins in living cells.

Research on G protein-coupled receptors (GPCRs) [1–5] plays a pivotal role in understanding cellular signaling and the development of therapeutic strategies. GPCRs are involved in a myriad of physiological processes and are the target for a large percentage of therapeutic drugs [5, 6]. Recent findings on a number of GPCRs have unveiled intricate molecular mechanisms that regulate receptor behavior, demonstrating distinct mechanisms of biased signaling alongside the modulation of conformational dynamics by ligand efficacy [7, 8]. These insights significantly enhance our understanding of the receptor’s functional versatility and its responsiveness to pharmacological interventions. The tendency of GPCRs to associate within the cell membrane has been clearly recognized, although the propensity for forming such complexes varies widely among major families and between distinct members of each family [9–13]. Similarly, the functional importance of such complexes has been shown to range from affecting ligand binding and/or downstream signaling events to having no apparent impact [14]. Class B GPCRs have been shown to be intermediate between the class C GPCRs, exhibiting required and often covalent dimerization, to class A GPCRs, with such complexes being mostly transient [10]. Of the class B GPCRs, the human secretin receptor (hSecR) is prototypic, most extensively studied, and best characterized [13, 15].

The secretin receptor has been shown to be capable of forming functionally important symmetrical dimers along the lipid face of transmembrane segment 4 (TM4), with this complex binding secretin with increased affinity and yielding a more potent signaling response than the secretin receptor monomer [16, 17]. The propensity for this receptor to form homodimeric complexes has been reported to be dependent on its concentration [18]. Recent fluorescence intensity fluctuation (FIF) studies of SecR revealed a mixture of not only monomers and dimers but also higher-order oligomers, such as tetramers and octamers, with the oligomer size increasing with receptor concentration [2]. Such high receptor concentrations could theoretically be generated during biosynthesis or in specific cellular compartments. Additionally, the FIF studies noted that treatment with secretin induced shifts in oligomeric size to higher orders. However, the nature and functional importance of these higher-order oligomeric complexes remain poorly understood.

The advent of technologies for cloning and mutating fluorescent proteins that can be fused to proteins of interest has greatly facilitated studies of protein-protein interactions in living cells [19, 20]. When one of the fluorescent tags acts as an acceptor (A) and another as a donor (D) of energy, it is possible to use **F**örster **r**esonance **e**nergy **t**ransfer (FRET) [21–23] to determine distances between parts of a molecule as well as the spatial distribution of protein complexes in living cells [24–27]. When more than one molecule or molecular complex are present in each pixel of a cellular image, an apparent FRET efficiency, $E_{app}$, is computed which incorporates fluorescence signals both from associated and unassociated proteins [21]. Developments in FRET theory [28, 29] and two-photon excitation microspectroscopy [21, 22] led to a quantitative FRET imaging method which allowed $E_{app}$ values to be determined for each image pixel after a single scan of the sample, from which histograms of $E_{app}$ could then be generated [23, 30]. From the analysis of the number and relative disposition of peaks within such $E_{app}$ histograms, or *FRET spectrograms* [23, 30], the quaternary structures (i.e., the geometrical shape and relative distances between protomers within an oligomer) have been determined, with molecular resolution, for several GPCRs in living cells [21, 31].

One of the critical parameters used in determining the geometry and distance between protomers is the orientation factor, $\kappa^{2}$, occurring between a FRET donor and an acceptor. Different donor/acceptor orientations can lead to a very wide range of $\kappa^{2}$ values (i.e., $0\leq \kappa^{2}\leq 4$), with its exact value(s) remaining unknown in FRET studies, due to random molecular motions that change the orientation of a fluorophore on a time-scale slower than the fluorophores excited state lifetime [32]. Uncertainties in $\kappa^{2}$ have so far dramatically limited the resolution of quaternary structure determination to molecular level only [30, 33], which prevents identification of binding interfaces between oligomers and any possible inter-relation between quaternary structure and conformational changes at the interface.

The growing availability of tertiary GPCR structures with atomic-level resolution [1, 34–37] and the development of powerful computational methods for performing molecular dynamics simulations [38–42] of the constrained translational and rotational diffusion of fluorescent proteins linked to the receptors of interest, has brought within reach previous proposals [23, 43] for determination of the quaternary structure with atomic resolution.

In this study, we present the design and implementation of such a method, dubbed FRET-MD spectrometry, and demonstrate its ability to determine, in living cells, the quaternary structure of hSecR, which exists as an equilibrium between monomers, dimers, and tetramers. We also identified key TM interfaces in mediating hSecR interactions. Our approach provided an amino acid-level picture of the previously proposed TM4:TM4 binding interface that occurs within dimers of hSecR in live cells and also identified a new interface occurring between TM5 domains, which appears to drive formation of hSecR tetramers and higher-order oligomers. In addition, this method provides the probabilities for dimer/oligomers to dissociate, which offer ideal handles on monitoring conformational changes that lead to oligomer association/dissociation, or vice versa. Taken together, these pieces of information suggest that the well-documented outward movement of TM6 on the cytosolic side, induced by ligand binding to GPCRs [8, 44, 45], facilitates the creation of a third binding interface (between TM6 and the first intracellular loop). This mechanism explains earlier observations that showed ligand binding to hSecR results in the stabilization of higher-order oligomers at the expense of monomers and dimers [2, 46]. In other receptors, this same outward movement could instead disrupt the formation of oligomers, as observed previously for the muscarinic acetylcholine receptor M1 [47], via e.g., steric clash.

The present research significantly enhances our understanding of GPCR oligomerization behavior and its connection to function, facilitating further exploration in the realms of molecular pharmacology and structural biology. In addition, the proposed technique is very general and can be applied to virtually any membrane protein in living cells.

## Results

### Instrument design and use

To determine the FRET efficiency, donor concentration, and acceptor concentration at each image pixel and thereby completely characterize the quaternary structure [23] of the highly dynamic [2] hSecRs oligomers in living cells, we needed a new two-photon optical micro-spectroscope that overcomes limitations of previous technology [21, 22], particularly their inability to extract the acceptor concentration in addition to the donor concentration and the FRET efficiency.

Firstly, we added a second femtosecond laser, tuned to 960 nm to excite both Ds (the green fluorescent protein variant GFP2) and As (yellow fluorescent protein, YFP), to the typical one that was tuned to 800 nm (to excite only Ds). Setting up the second laser beam to trail the first one by just 100 ms allowed successive scans of each sample voxel at the two different wavelengths faster than molecular diffusion requires to fully scramble the oligomeric makeup of that voxel. The 960-nm scan was applied first to the sample to avoid switching Ds to a different state by excitation at 800 nm before measuring them at 960 nm [31, 48].

Secondly, to increase the normally low image acquisition speed of two-photon micro-spectroscopes with point-scan capabilities [22], two spatial light modulators (SLMs) were employed to separately shape each of the two light beams into 8 by 4 arrays of beamlets at the sample plane, as described in the Methods section. By raster scanning these two arrays of 32 beams against the samples, fluorescence images were acquired of hSecR-GFP2 and hSecR-YFP either singly expressed or co-expressed in live HEK-293 cells. The single expression provided elementary spectra of GFP2 and YFP [4]. These were used as described previously [49] to spectrally unmix the composite emission spectra for every pixel in the fluorescence images of cells co-expressing SecR-GFP2 and hSecR-YFP. Two sets of two-dimensional (2D) fluorescence intensity maps were obtained. The first set contains the fluorescence intensities of donors in the presence of acceptors ($k_{1}^{DA}$) and acceptors in the presence of donors ($k_{1}^{AD}$) for the first excitation wavelength (960 nm), designated with index “1” herein (see Figure 2a–c). The second set, $k_{2}^{DA}$ and $k_{2}^{AD}$, represents the fluorescence intensity of donors in the presence of acceptors and acceptors in the presence of donors, respectively, for the second excitation wavelength (800 nm), denoted by ‘2’.

The $k_{2}^{DA}$ and $k_{2}^{AD}$ maps (Figure 2b, c) were used to calculate the apparent FRET efficiency, $E_{app}$, of individual image pixels containing an as yet undetermined number of oligomers of different sizes [31], employing Equation (2) in the Methods section. The $k_{2}^{DA},\;k_{2}^{AD}$, and $k_{1}^{AD}$ maps (see Figure 2a–c) were used to determine the donor and acceptor concentration for each pixel in the fluorescence images by a set of equations derived previously [29] as described in Supplementary Methods SM4. The total receptor concentration per image pixel was determined as the sum of the donor and acceptor concentrations.

### Generation and analysis of FRET efficiency spectrograms

Each cell in the unmixed images was demarcated with a hand-drawn polygon, and a computer program then generated a second polygon positioned at the intracellular boundary of the plasma membrane. The space between the two polygons, comprising the cell membrane, was designated as a region of interest (ROI), as shown in Figure 2. To capture molecular distribution inhomogeneities, each ROI was divided into smaller segments that, given the receptor expression levels used, contain just a few to a few tens of oligomeric structures (see Supplementary Methods section SM4). Histograms of pixel-level $E_{app}$ for three consecutive ROI segments colored in cyan in Figure 2d are shown on the first row of histograms in that panel. As it is seen, the histograms exhibited two or more sharp peaks corresponding to frequently occurring oligomeric configurations within the pixels of a given segment. The distribution of peak positions varied from segment to segment, influenced by segment-level receptor concentration and [D] to [A] concentration ratio. Variation in [D]/[A] was deliberately enhanced by co-transfecting HEK-293 cells with plasmids encoding donor- and acceptor-tagged receptors in four different ratios (1:1, 1:2, 1:3, 1:5). The *$E_{app}$* values associated with the two highest peaks identified in each ROI segment, using an algorithm described in Supplementary Methods section SM7, were used to assemble *FRET spectrograms*, or meta-histograms of peak positions [2, 31], as described next. The total protomer concentration was also determined for each $E_{app}$ histogram peak from the median value of concentration across the pixels associated with the peak.

Signals originating from oligomers manifest themselves as clusters of pixels with similar FRET efficiency values due to the Point Spread Function (PSF) of the microscope, and they lead to peaks in the $E_{app}$ histogram. It is also possible for pixels scattered throughout an ROI segment to show similar $E_{app}$ values simply due to noise; this may inadvertently lead to a peak in an $E_{app}$ histogram. To increase the frequency of peaks originating from true oligomers relative to those caused by random noise, we used an approach dubbed “contour-guided re-segmentation” of the ROI (see Supplementary Methods SM5), according to which the starting point for each segment was shifted along the ROI curve by about one-quarter of the original segment lengths, thereby generating a new set of segments from the same ROI [2, 31]. The re-segmentation was repeated after shifting the starting point three times for each ROI drawn. The results of two such re-segmentations are shown by red and green histogram plots in Figure 2d, alongside those corresponding to the original segment set (depicted with cyan lines). FRET efficiency peaks originating from clusters of adjacent pixels, due to the PSF of real oligomers, should appear one or two more times in subsequent re-segmentations – compare the peaks occurring in the cyan-colored to those in the red- and green-colored $E_{app}$ histograms in Figure 2d. Conversely, peaks caused by random noise scattered throughout a given segment are unlikely to appear again in the re-segmented ROI.

### Overview of the quaternary structure determination process

To extract the quaternary structure information contained in the FRET spectrograms, we employed a novel approach that fit the FRET spectrograms with FRET efficiency values obtained from molecular dynamics (MD) simulations of fluorescently tagged hSecR oligomers embedded within a plasma membrane environment. These simulations allowed fluorescent proteins to diffuse under the constraints of their linkers, just as they are believed to do within their cellular milieu. For each time point, from the positions (r) and relative orientations ($\kappa^{2}$) of the fluorophores attached to each protomer within the oligomer, we computed $E_{app}$. We then fit the experimental FRET spectrograms with distributions of MD simulations-based $E_{app}$ histograms, which allowed us to determine the most likely oligomeric structure and the dissociation probabilities of protomers within the oligomeric configuration, as detailed below.

Thirty different configurations (see Supplementary Table 2) for the largest oligomeric unit investigated – the tetramer – were generated based on the orientation of the inter-protomeric TM interfaces relative to one another. Each tetramer was created by duplicating a dimer structure and arranging the duplicates so that the van der Waals surfaces of the closest contact points between the dimers did not significantly overlap at the ‘tetramer’ interface, as will be shown below. All tetramer structures had three contact interfaces: two from the TM domains in the dimer interface and one from those in the tetramer interface. The roles of the TM domain pairs in a tetramer were interchangeable, allowing for the formation of a “conjugate” tetramer. By including the original tetramer and its conjugate as part of the whole model, we obtained 15 different tetrameric model candidates. To speed up the process of identifying the quaternary structure model that best fits the FRET spectrogram data, we first used structures that were only equilibrated. These initial equilibration steps involved subjecting the tetramer models to a short molecular dynamics (MD) simulation equilibration routine to allow the system to reach a stable configuration without performing a full production run. The FRET efficiency of each equilibrated structure was computed and compared to the measured distribution of FRET efficiencies seen in the FRET spectrogram data. This preliminary equilibration was sufficient to observe general trends and interactions, enabling us to screen for the most promising and least promising tetrameric configurations quickly. Based on these preliminary results, we identified the best and worst candidate structures for further, more detailed simulations. Of those, eight (i.e., two times four conjugate pairs) were fully simulated, leading to the identification of the four most likely quaternary structures, which are listed in Supplementary Table 3 and further investigated as described in our exposition below.

### Molecular dynamics simulations of tetramers in the plasma membrane

MD simulations were performed on molecular structure files consisting of an inactive hSecR attached to a GFP2 fluorescent protein (See Methods and Supplementary Method SM8). The inactive conformation of hSecR was generated through simulations starting from the known active structure file for hSecR [1]. We tracked the movement of the TM6 domain until its orientation resembled that of other GPCRs with known inactive conformations (See Supplementary Figure 3) [44, 45]. Using that structure, we generated the four different dimers shown in Figure 3. To create each dimer, two identical hSecR-GFP2 monomers were positioned in close proximity to each other (see Methods). The residues of closest contact were located on the same TM domain in both monomers and were designated as the ‘dimer interface’ (Figure 4a). No specific preference was given to which residues were closest to one another, except for one initial dimer structure (“TM4 hydrophobic” in Figure 3), which was inspired by previous studies [16, 17].

To estimate the FRET efficiency of a particular oligomer structure, it was essential to keep the structure near the chosen (initial) contact interfaces throughout the simulation. This allowed us to obtain the spatial trajectories and orientations of the fluorophores needed for computing the FRET efficiency, while keeping the computing time down by several orders of magnitude, compared to the alternative of simulating freely diffusing protomers and then selecting for those that have been in contact. For this, we capitalized on what has been perceived sometimes as a limitation of the MARTINI 2 force field, that it inadvertently enhances interactions among membrane-embedded proteins compared to more polar proteins [50]. To further enhance this effect, we used much higher protomer concentrations (~7,000 protomer/μm^2^) than those at which hSecR forms monomers [8] or those in the present experiments.

### Computing FRET efficiency for each dimer/tetramer from MD simulation trajectories

To compute the apparent FRET efficiency values for a particular oligomer structure, we determined for different time points the distance between the centers of mass ($r_{a,b}$) and the orientation factor ($\kappa_{a,b}^{2}$) between the transition dipole moments (as defined by Equation SM29) of the chromophores attached to the protomers. These calculations began 100 ns into each simulation and occurred at 100-ps intervals until the end of each 1-μs simulation. We then used Equation (4) of the Methods section to calculate the apparent FRET efficiency for the entire complex, $E_{n,k,q,f}^{MD}$, for each frame. This was done for all eight 1-μs long simulations. The $E_{n,k,q,f}^{MD}$ values were averaged over all frames across eight separate simulation runs.

For a single tetramer structure, there are 16 different configurations corresponding to different placements of donors and acceptors at each of the protomer locations. Among these configurations, two involve either all donors or all acceptors, resulting in no FRET. The other fourteen configurations, where donors and acceptors are mixed, produced non-zero apparent FRET efficiency values; those 14 configurations are illustrated in Figure 4d. We used the distance and $\kappa^{2}$ data obtained from the MD simulations to calculate apparent FRET efficiency values for each of the 14 possible configurations within a given MD-simulated tetramer structure (see Supplementary Table 3 for a list of all 8 MD-starting tetramer structures). All of the tabulated FRET efficiency values were then inserted into a theoretical oligomer mixture model, described below, in order to identify the quaternary structure model that best fits the peaks of the experimental FRET spectrogram.

### Oligomer dissociation modifies the predicted FRET efficiency distributions

Our model does not presuppose stable tetramers, but rather allows for association/dissociation among hSecR protomers leading to a mixture of monomers, dimers, trimers, and tetramers. In this framework, data fitting uses information from MD simulations corresponding to two distinct MD-starting tetramer structures (see Supplementary Table 3). For example, envision a pair of conjugate tetramer structures established through interactions between TM domains A and B. One structure exhibits two contact interfaces at TMA domains and an additional one at TMB, designated as TMA/TMA:TMB/TMB. Conversely, the type denoted as the TMB/TMB:TMA/TMA conjugate tetramer showcases two contact interfaces at TMB and an additional one at TMA. Importantly, allowing interactions at these three interfaces enables the formation of higher-order oligomers. The conjugate tetramers may be regarded as originating from the same higher-order oligomer dissociating along different interfaces (see Supplementary Figure 5).

Our fitting model incorporates sixteen potential dissociation states, each comprising mixtures of monomers and oligomers of various sizes, as illustrated in the bottom panel of Figure 4d. The percentage of time the protomers exist in each of the dissociation states was derived from the probability of dissociation of the TM domains comprising TMA ($P_{A}$) and the probability of dissociation of the TMB domains ($P_{B}$) using the relations shown in Figure 4d. The dissociation probabilities of the TM domains $P_{A}\;P_{B}$ and the FRET efficiency values obtained from the MD simulations for each of the dimers, trimers, and tetramers were used to calculate the average FRET efficiency of an ensemble of oligomers ($E_{mix,k,q}^{MD}$) for each oligomeric configuration, q, according to Equation (5). The fourteen $E_{mix,k,q}^{MD}$ values calculated for each configuration (Figure 4d top) were used as the respective centers of fourteen Gaussian functions [see Equation (6)] whose sum was used to fit the experimental FRET spectrogram. Note that oligomers larger than four, whose influence on the FRET spectrogram would manifest most notably in the tails of the FRET spectrogram [51], were ignored for simplicity.

### The hSecR quaternary structure

Using a systematic fitting procedure that adjusted the dissociation probabilities, $P_{A}$ and $P_{B}$, as well as the amplitudes and standard deviations of the Gaussian functions for all four oligomer structure candidates (corresponding to the TM domain conformation pairs listed in Supplementary Table 3), we found the lowest fitting residuals corresponding to each structure (see Supplementary Figure 6). Among those, the TM domain conformation pair mediated by interactions at the portion of the TM4 domain within the hydrophobic region of the membrane as well as the TM5 domain provided the best overall fit (see Figure 5, panels a-c).

To ensure the reliability of the best fit to the full FRET spectrogram and rule out its dependence on noisy data points, we employed a ‘bootstrapping’ procedure for resorting and analyzing the data. This approach involved generating 24 different FRET spectrograms, each constructed by randomly selecting a subset of the $E_{app}$ histogram peak values and constructing a new FRET spectrogram from the corresponding subset. These FRET spectrogram sub-samples underwent the complete systematic fitting procedure described above. Supplementary Figure 7 displays 24 different FRET spectrogram sub-samples and the overall best fit to each of them. We counted the number of times a particular TM domain conformation pair resulted in the best fit of a sub-sampled FRET spectrogram, which allowed us to assess the variability in the model’s estimates (see Supplementary Figure 8).

Among all the sub-sampled FRET spectrograms, the TM domain conformation pair characterized by interactions at the hydrophobic portion of the TM4 domain and the TM5 domain achieved the best fit most frequently (18 out of 24 times). This conformation pair also yielded the best fit when fitting the full FRET spectrogram, as described in the previous paragraph. The higher frequency of this structure in our bootstrapping analysis is indicative of the robustness of the overall best fit, and ultimately that this model most likely represents the hSecR tetramer’s structure (see Figure 5, panels d and e). The second most frequently observed pair (fitting 5 out of 24 sub-sampled FRET spectrograms) also involved TM domains 4 and 5, but with interactions in the TM4 domain that occurred primarily at the extracellular part of the membrane. While we cannot discard the possibility that this so-called hydrophilic TM4/TM4 tetramer may occur (with a frequency of 6 out of 24, or 25% of the time), we take its hydrophobic version (which provided the best fit 75% of the times) as the most likely quaternary structure of hSecR. The dimeric interface of this structure also agrees with previous investigations [16, 17].

The dissociation probabilities $P_{A}$ and $P_{B}$, determined from fitting the experimental FRET spectrogram data with the MD simulated FRET efficiency values, describe the likelihood of dissociation of individual protomers at the specific binding interfaces within the oligomer and measure the oligomer stability. The average dissociation probabilities for the best-fitting tetramer structure were computed by averaging the probabilities obtained from the 18 (out of 24) instances when the given structure achieved the best overall fit to a sub-sampled spectrogram (see the plots labeled “TM4 hydrophobic & TM5” in Supplementary Figure 7). As seen in Figure 5 and Supplementary Table 4, the average dissociation probabilities at the TM4/TM4 hydrophobic and TM5/TM5 interfaces exhibited similar values (given their respective error bars), implying similar binding strengths at the two identified interfaces. These probabilities were used to calculate the average fraction of monomers, dimers, trimers, and tetramers (see Supplementary Table 5) using the formulas in Figure 4d. The result (see caption to Figure 5) was that the hSecR population consists primarily of monomers, with progressively smaller fractions forming dimers, trimers, and tetramers. The corollary to this is that, at significantly higher concentrations, dimeric or tetrameric species dominate. These observations align with our previous findings on hSecR interactions using fluorescence intensity fluctuation (FIF) spectrometry [2].

We closely monitored the residues that were consistently ‘in contact’ with residues from other protomers within the tetramer for the duration of the MD simulations and computed a ‘residue-level contact frequency,’ which is similar to the ‘domain-level contact frequency’ (see Methods). Two residues from different protomers were considered to be in contact if situated within 5 Å of one another. Figure 6a shows a 3D ribbon representation of the tetramer structure featuring residue-level interactions involving the conformation pair consisting of a TM4/TM4 hydrophobic interface and a TM5/TM5 interface, with each of the four protomers within the tetramer highlighted by a unique color.

The interaction interface formed by TM4 domains is highlighted in Figure 6a within a black rectangular dashed region. A zoomed-in view of this interface is displayed in the inset to the right of the black dashed rectangle, providing a map of the residue pairs with the highest overall contact frequency, labeled with their residue number and single-letter amino acid identifier. Figure 6a only shows one of the two conjugate tetramer structures comprising the TM domain conformation pair. However, the pairs of residues highlighted as forming the highest contact frequencies were identified by calculating their contact frequencies from MD simulations of this starting structure and its conjugate pair (see Supplementary Table 3). The most frequently occurring residue pairs in contact on the TM4 domains are ALA246/ILE247 (0.49), HIS257/HIS257 (0.51), and ALA239/ALA239 (0.49), with the values in parentheses representing a pair’s computed contact frequency. The first and third residue pairs, interacting in the hydrophobic core of the membrane (ALA246/ILE247 and ALA239/ALA239), most likely involve Van der Waals forces, which are significant in a densely packed membrane environment. The small side chain of alanine allows for steric complementarity, enhancing the stability of interactions with the bulkier side chain of isoleucine (ILE247) and between alanine residues (ALA239). In the extracellular region, histidine-histidine (HIS257/HIS257) interactions are primarily driven by π-π interactions due to the aromatic nature of the imidazole ring in histidine. Collectively, these interactions appear to be crucial in supporting the stability of the TM4/TM4-mediated binding interface.

The interaction interface formed by TM5 domains is highlighted in Figure 6a using a red dashed rectangle; the inset to the left of the structure represents a magnified view of this region in the tetramer. The pairs of residues most frequently in contact on the TM5 domains are LEU283/LEU286 (0.39). The nonpolar side chains of these leucines may engage each other via Van der Waals interactions, which are very short-range forces. Therefore, for this binding interface to form, either the receptor density must be high, or some other factor must first bring the two surfaces together, and then LEU/LEU interactions may participate in stabilizing the quaternary structure formed.

Figure 6b shows a three-dimensional surface representation of hSecR tetramer TM domains, using residue-specific coloration to visualize residue contact frequency. The residue-level contact frequencies displayed in Figure 6b are normalized to 1 by dividing the contact counts for each residue by the highest contact count observed across any residue throughout the simulation. The lower portion of each panel displays the actual molecular structure, while the upper part shows the corresponding exploded view.

## Discussion

By combining FRET spectrometry with MD simulations, we successfully identified two key TM domains, TM4 and TM5, that drive interactions between hSecR in living cells. Previous mutagenesis experiments targeting specific residues (GLY243 and ILE247) in TM4 [16] highlighted the significance of the TM4/TM4 binding interface as a primary driver of interactions between hSecR protomers within dimers. Those mutations resulted in a decrease in bioluminescence resonance energy transfer (BRET) signal occurring between hSecR, and, concomitantly, a reduction in hSecR’s ability to stimulate intracellular cAMP. This functional effect underscores the physiological relevance of hSecR oligomerization. To further refine those observations, we conducted similarly designed experiments using FRET (instead of BRET) between donor-tagged and acceptor-tagged receptors expressed in equal amounts. To obtain subpopulations of cells in which the donor and acceptor molecules occurred on average in equal amounts in both the mutant and wild-type cells, we filtered much larger data sets based on the concentrations computed from FRET measurements. When determining the average FRET values for both wild-type hSecR and mutant hSecR (GLY243ALA, ILE247ALA) from filtered ROI segments, the observed reduction in the average FRET efficiency was significant but small, i.e., less than 10% (Supplementary Table 6).

The fact that the average FRET (in this study) and BRET signals (in the previous study) remained significantly above zero after mutating a number of residues on the TM4 domain suggests the existence of additional interaction interfaces within hSecR, possibly contributing to the formation of higher-order oligomers. This was indeed confirmed by our detailed FRET spectrometry analysis presented above. Attempts to fit the measured FRET data using a model consisting only of a mixture of monomers and dimers interacting at the TM4 interface (see Supplementary Figure 9a) or TM5 interface (see Supplementary Figure 9b) resulted in a rather poor fit compared to a model that incorporates the presence of tetramers. Using the latter model, not only have we confirmed the results of the previous assays that identified the TM4/TM4 interface, but also uncovered the second oligomeric interface (TM5/TM5) within the secretin receptor oligomer (hSecR).

We also found that the average dissociation probabilities at TM4/TM4 and TM5/TM5 interfaces from such a complex were approximately equal, indicating comparable interaction strengths. Based on the obtained dissociation probability values, we observed that, at lower expression levels, the population of hSecR predominantly existed as monomers, with smaller proportions of dimers, trimers, and tetramers. This is in agreement with a previous study by some of us which was based on fluorescence intensity fluctuation spectrometry, which helps validate the accuracy and utility of the present methodology [2].

Additionally, our MD simulations enabled the identification of the most frequent residue pairs involved in interactions at the oligomeric interfaces, as depicted in Figure 6. Through detailed analysis, we identified the type of forces governing these interactions, revealing a combination of non-covalent forces, including π-π interactions and Van der Waals interactions for the TM4/TM4 binding interface, and Van der Waals interactions for the TM5/TM5 binding interface.

Our previous studies [2, 46, 52] have shown that secretin binding to hSecR promotes the formation of higher-order hSecR oligomers at the expense of monomers and lower-order oligomers. Ligand binding to class B1 GPCRs (which hSecR belongs to) is known to induce primarily an outward movement of the cytosolic part of TM6 [8, 44, 45], as also seen from the comparison of the crystal structure of the ligated active hSecR to our MD simulations of hSecR when ligand is removed, shown in Supplementary Figure 3. While this movement appears to have minimal impact on the binding interfaces identified in this work, we wanted to see if it may affect oligomerization. For this, we reassembled the hSecR tetramer comprising the TM4/TM4(hydrophobic) and TM5/TM5 binding interfaces from activated protomers (in which the cytosolic part of TM6 points outward), equilibrated the reassembled structure, and compared this structure to the equilibrated tetramers consisting of inactive protomers (see Figure 7). Remarkably, the activated hSecR tetramer not only accommodated the outward movement of TM6 but also led to the creation of a third binding interface between TM6 and the first intracellular loop (ICL1) of hSecR, which should further stabilize the tetramer.

The residues in close proximity on TM6 and ICL1 are labeled in Figure 7 and include histidine (HIS150) on ICL1, and alanine (ALA320), arginine (ARG321), and leucine (LEU324) on TM6. Histidine is particularly versatile in its role in molecular interactions due to its unique structure, allowing it to participate in various classes of interactions, depending on the surrounding amino acids and pH environment. Based on the average nearest-neighbor amino acids identified from multiple MD equilibration runs of the liganded (i.e., active) hSecR tetramer, we conclude that the histidine on the ICL1 of one SecR protomer is most likely involved in a π-cation interaction with the nearby arginine residue on the TM6 domain of a neighboring protomer. This interaction appears to further stabilize the tetrameric structure mediated by (the presumably weak) TM5/TM5 binding interface and promote the association into even larger structures along the same binding interfaces, as observed previously [2, 46, 52].

Taken together, the above interactions nicely explain previous experimental findings [2, 46, 52] that the association/dissociation equilibrium of hSecR shifts toward larger oligomers both at high concentrations – which bring protein domains close enough to form Van der Waals bonds – and following treatment with agonist ligand – which locks the TM6 domain in a conformation that leads to formation of an additional binding interface.

It is interesting to speculate that, when applied to different receptors, the same quaternary structure with the same outward movement of TM6 could instead disrupt the formation of higher-order oligomers (e.g., via steric clash or electrostatic repulsion). For example, such disruption in oligomer formation has been observed previously for the muscarinic acetylcholine receptor M1R [47]. Notably, M1R has a very large third intracellular loop (>100 amino acids) [53, 54] which supports the steric clash hypothesis, although a definitive conclusion in that regard will need to be deferred until the M1R oligomer structure and its actual binding interfaces are determined experimentally.

In conclusion, our new FRET-MD spectrometry approach provided a robust platform for investigating the oligomerization of G protein-coupled receptors with known tertiary structures. The method could be used in principle to investigate structures of any membrane protein.

## Methods

### Instrumentation

#### Description of the two-photon micro-spectroscope with dual-wavelength excitation

**Figure 1.**
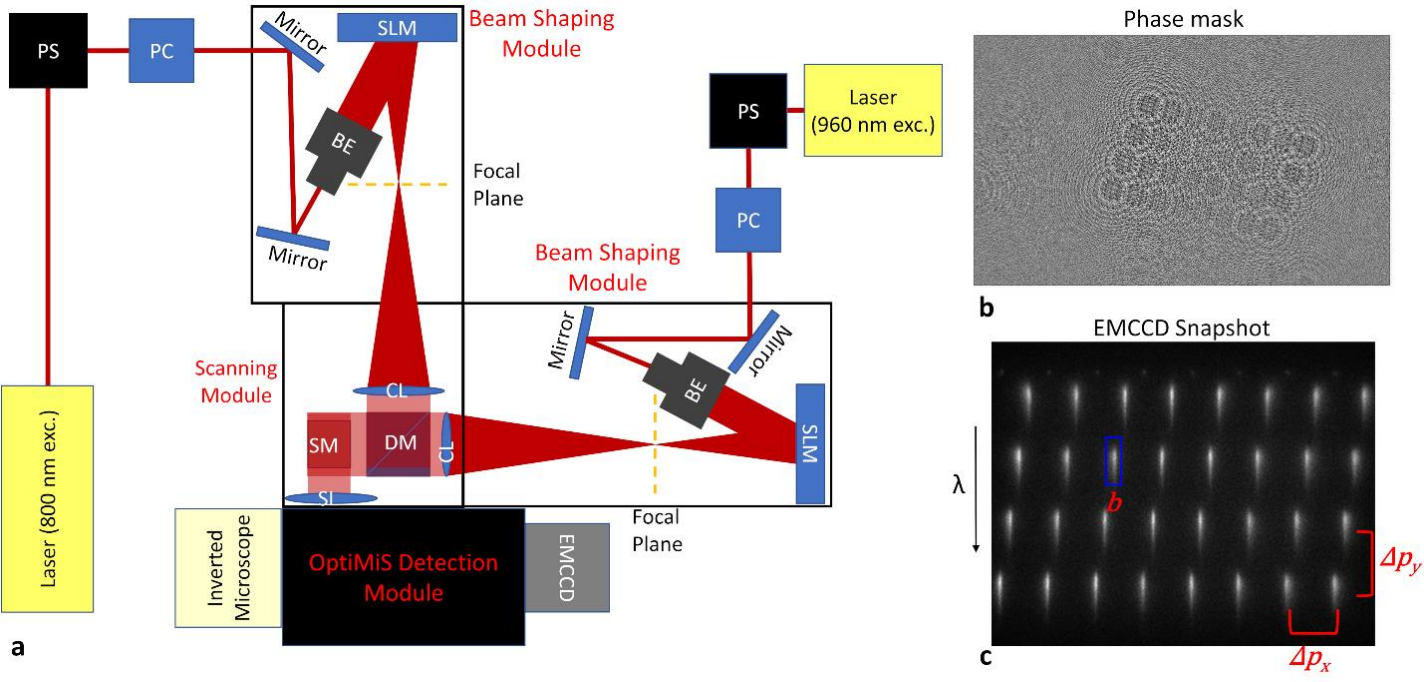
Dual excitation spectrally resolved optical microspectroscope setup and raw data. **(a)** Schematic of the optical microspectroscope: SLM, Spatial Light Modulator; SM, Galvanometric Mirrors; CL, Collimating Lens; SL, scanning lens; DM, Dichroic Mirror; PS, Periscope; BE Beam Expander; PC, Power Control Module. **(b)** SLM-generated phase mask resulting in each laser beam being patterned into an 8×4 array of excitation voxels. **(c)** Fluorescence emission pattern of a uniformly fluorescing sample of uranine excited by the laser beam array generated using the phase mask from (a). The blue rectangle indicates the region of pixels on the EMCCD used to reconstruct the micro-spectroscopic data set. These snapshots are reconstructed into a 3D data cube, where each slice represents the 2D fluorescence emission at a different wavelength. The columns of each row are summed to obtain a fluorescent intensity value for each pixel in a single wavelength channel of the reconstructed data cube, with each row corresponding to emission in a different wavelength band.

The custom two-photon optical micro-spectroscope (See Figure 1 for diagram) developed as part of this study is comprised of a Zeiss Axio Observer inverted microscope stand equipped with an infinity-corrected water immersion objective (63×, NA=1.2; Carl Zeiss Microscopy), a cooled electron-multiplying CCD (EMCCD) camera (iXon Ultra 897, Andor Technologies), and an OptiMiS detection head which is equipped with a transmission grating that projects the spectrum onto an EMCCD camera [22]. The spectral bandwidth of the wavelength channels ranged from 450 nm to 600 nm with a spectral resolution of 12 nm. A MaiTai^®^ Deep See^™^ (Spectra-Physics) ultrafast laser with automated dispersion compensation and a tunable center wavelength was used for fluorescence excitation at 800 nm. A separate MaiTai^®^ (Spectra-Physics) ultrafast laser was used for fluorescence excitation at 960 nm. Each of the two laser beams were passed through separate power control modules and custom-made beam shaping modules and were combined using a dichroic mirror, which will be described in more detail below. The spatial light modulator (for laser beam shaping), optical scanning head (for laser beam scanning), and EMCCD camera used for image acquisition were controlled by the same computer using in-house custom software written in C++.

#### Patterning and combination of two separate excitation lasers

**Figure 2.**
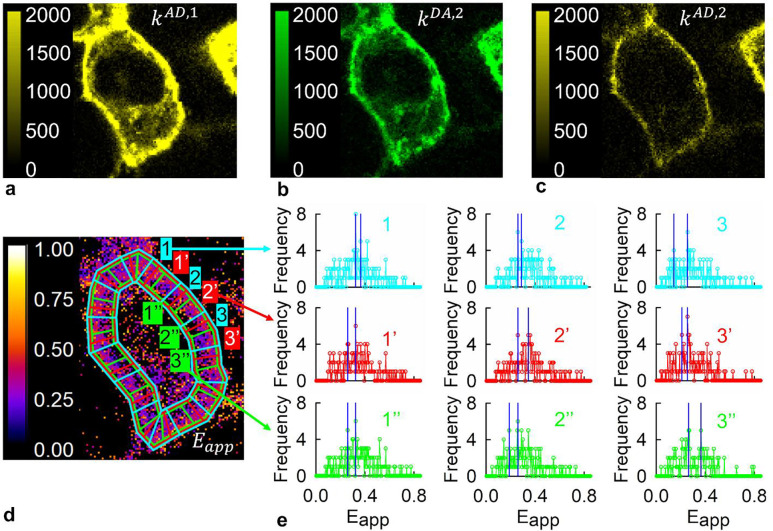
Illustration of the image processing workflow in advanced FRET spectrometry. **(a,b,c)** Representative 2-D maps obtained through pixel-level spectral unmixing of fluorescence images from living HEK-293 cells co-expressing GFP2 and YFP-tagged hSecR. The acceptor emission intensity at the first excitation wavelength (k^AD,1^) as well as donor (k^DA,2^) and acceptor(k^AD,2^) emission intensity at the second excitation wavelength are shown. **(d)** The 2-D apparent FRET efficiency map ($E_{app}$) was computed from the pixel-level values of k^DA,2^ and k^AD,2^, as described in the Methods section. Region of interest (ROI) segments were generated using a contour-guided multiple segmentation process (Supplementary Methods section SM5); three representative sets of segments were projected over a cell (shown in cyan, red, and green colors). **(e)** Examples of $E_{app}$ histograms for nine different segments using a bin size of 0.005. The cyan-colored histograms in the top row are derived from three segments delineated by cyan-colored lines (labeled 1, 2, and 3) in (d), the red-colored histograms in the middle row correspond to three re-segmented segments (labeled 1’, 2’, and 3’ and outlined with red-colored lines), and the green-colored histograms in the bottom row correspond to the three re-segmented segments outlined with green lines (labeled 1”, 2”, and 3”). An automated peak selection method (see Supplemental Methods section SM6), identified the two most significant peaks in each histogram, indicated by vertical blue lines. The selected peak locations for each of the histograms are listed in Supplementary Table 1.

**Figure 3.**
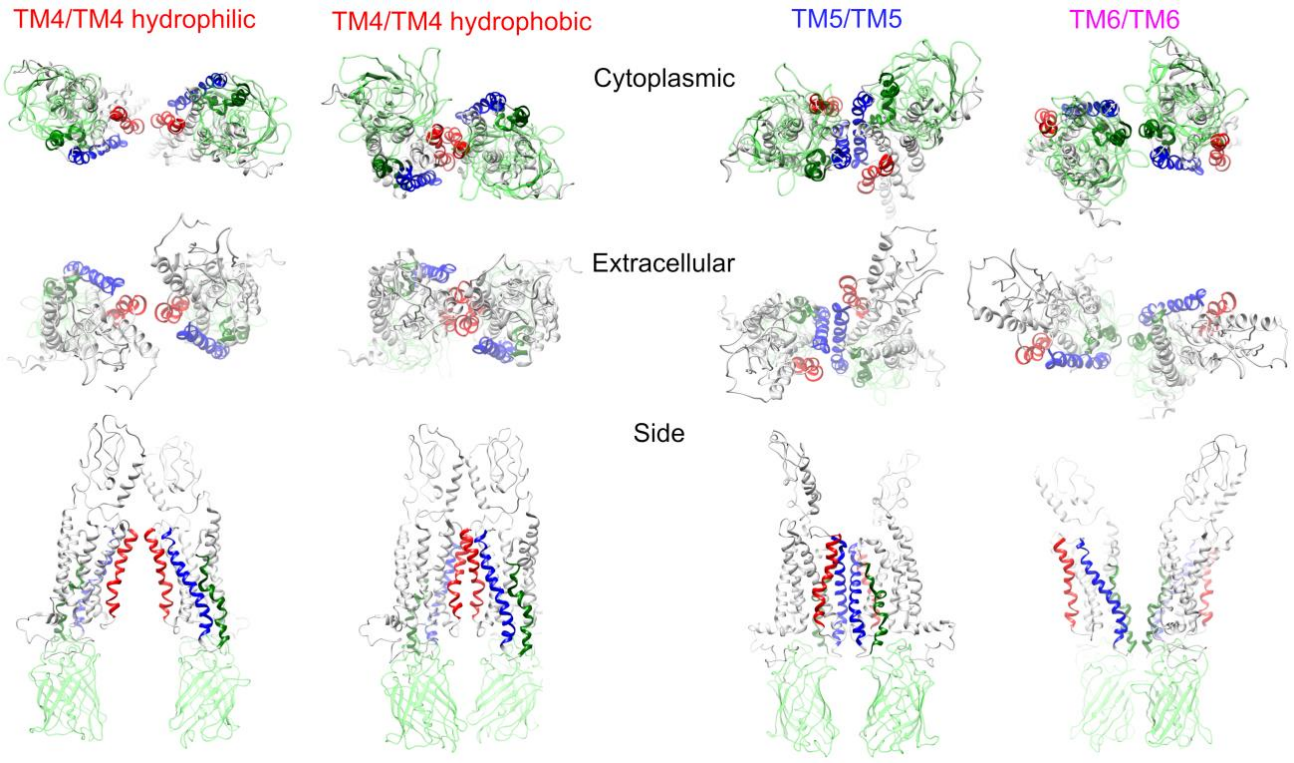
Construction of starting oligomeric configuration of hSecR-GFP2. Three distinct dimer orientations were generated by rotating each protomer around the axis perpendicular to the cell membrane. This positioning ensured that a different homologous pair of transmembrane (TM) domains faced each other at the dimer interface for different starting configurations, specifically TM4/TM4, TM5/TM5, and TM6/TM6. A fourth dimer orientation (TM4/TM4 hydrophobic) was generated by rotating each protomer around this same axis perpendicular to the cell membrane but also tilting the protomers in opposite directions around an axis in the plane of the cell membrane. The hSecR dimer structures are presented from cytoplasmic, extracellular, and side views, with domains near the contact interfaces highlighted in respective colors. The complete list of the receptor domains involved in the four tetramer structures shown in this figure is provided in Supplementary Table 3.

**Figure 4.**
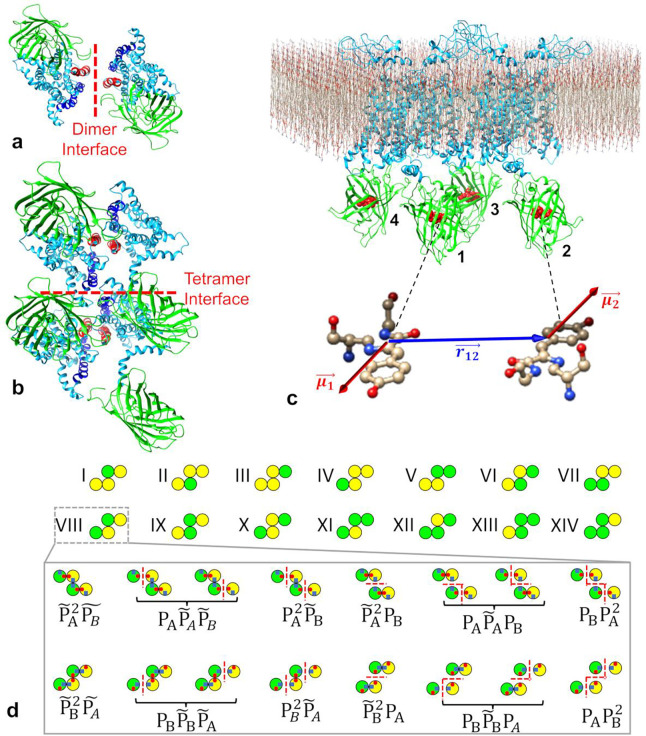
Determining complex level FRET efficiency values from MD simulated hSecR structures. **(a)** A dimer structure consisting of hSecR (shown in cyan color) tagged with a fluorescent protein (shown in green color) with interaction driven by residues in the TM4 (shown in red) interface. **(b)** A tetramer structure constructed from two copies of the dimer shown in (a). The interaction between the dimers is driven by residues in the TM5 (shown in blue color) interface. **(c)** The tetramer structure inserted into a plasma membrane. The transition dipole moments ($\mu_{1}$ and $\mu_{2}$ for protomer 1 and 2, respectively) and distance ($r_{12}$) between centers of mass of two chromophores (depicted as red-colored spheres within fluorescent proteins) for each possible pair of protomers in an oligomeric structure were calculated from each frame of eight 1 μs long MD simulations. The orientation factor (see Supplementary Equation 29) and distance values were then used to calculate the FRET efficiency of the entire oligomeric complex for each frame of the eight MD simulation trajectories. **(d)** Top panel, 14 different configurations of the tetramer, corresponding to different placements of donors (green circles) and acceptors (yellow circles) at the available protomer locations. Lower panel (inside the rectangular box), probabilities of occurrence of each of 16 possible dissociation states of the oligomer resulting from dissociation at one of three possible interfaces. These depend on two parameters, the dissociation probability ($P_{A}$) of protomers interacting via the TM4 domain and the dissociation probability ($P_{B}$) of protomers interacting via the TM5 domain. Here, ${\tilde{P}}_{A}=1-P_{A}$ and ${\tilde{P}}_{B}=1-P_{B}$.

Two laser beams were individually shaped using beam shaping modules (BSMs), which first expanded the beams to a 1/e^2^ diameter of ~10 mm, using a fixed length Galilean beam expander (Thorlabs, Inc), and then directed them to a phase-only spatial light modulator (P1920–1152-HDMI Nematic SLM System, Meadowlark Optics) consisting of a 1920 × 1152 array of pixels and 9.8 μm spacing between each pixel. SLMs were utilized to transform the incident Gaussian laser beam into an array of focused points by introducing a specific spatially varying phase pattern across the wavefront of the incident beam. For the measurements described in this manuscript, each of the laser beams passing through one of the BSMs was shaped into an 8×4 array of beamlets by projecting a phase-mask onto the SLM emulating a 2D microlens array (See Figure 2b) using a procedure described in detail in the Supplementary Methods Section SM1. The angle of incidence between the surface normal of the spatial light modulator (SLM) and the propagation direction of the laser beam was 5°.

After reflecting from the surface of the SLM, the beams were collimated by achromatic doublets (AC254–200-B-ML). The spacing between the SLM surface and the collimating lens (CL in Figure 1) was set to be equal to the sum of the focal lengths of the collimating lens (f=200 nm) and virtual lenses (f=315 nm) of the phase-mask projected onto the SLM. The patterned laser beams were combined using an ultra-flat long pass dichroic mirror (DM in Figure 1; T900lpxxrxt-UF2, Chroma Technology Corp) and then directed onto the same pair of galvanometric mirrors (SM in Figure 1), enabling synchronous scanning in two orthogonal directions in the sample plane. Additional details on the formation of the array of beams, image acquisition and reconstruction are given in the Supplementary Methods Sections SM2 and SM3.

A full set of micro-spectroscopic images, which consisted of two sets of images (one set for each excitation wavelength) each containing 445 × 260 pixels and 20 emission wavelengths per pixel, was acquired by simultaneously scanning the two patterned laser beams across the sample. Each location in the sample was first excited by the laser tuned to 960 nm, followed by excitation of the laser tuned at 800 nm. For this reason, the 960 nm and 800 nm excitation wavelengths are referred to in the rest of the paper as the 1^st^ and 2^nd^ excitation wavelengths, respectively.

The total acquisition time for a full set of micro-spectroscopic images was ~26 seconds. The dwell time of the laser beam in each excitation voxel was set to 7.5 msec/pixel. The average power per excitation voxel was 2.3 mW. The raw data were reconstructed into 2D fluorescence images using a procedure described in the Supplementary Methods section SM3.

### FRET measurements and data pre-processing

#### Cell Sample preparation

Human secretin receptor constructs, featuring carboxyl-terminal tags of either green fluorescent protein 2 (GFP2) or yellow fluorescent protein (YFP), were ligated into the pcDNA3 expression vector as described previously [16]. These constructs, denoted as SecR-GFP2 and SecR-YFP, respectively, mimicked the functionality of the wild-type secretin receptor[16].

Human embryonic kidney cells (HEK-293) were cultured in T-25 flasks with 5 ml of high glucose Dulbecco’s Modified Eagle Medium (DMEM, Life Technologies), supplemented with 10% fetal bovine serum, 2mM L-glutamine, 100 μg ml^−1^ penicillin, and 0.1 mg ml-1 streptomycin. Maintained at 37°C in a humidified environment with 5% CO_2_, cells were lifted from the T-25 flask and seeded at ~40% confluency onto either 35 mm glass-bottom dishes (Cellvis) or Lab-Tek eight-well-chambered cover glasses (Thermo Fisher Scientific); both dish types were pre-coated with Poly-D-Lysine (Sigma-Aldrich).

After 24–48 hours of incubation, cells were transfected with plasmids encoding SecR-GFP2 and SecR-YFP. The transfection mixture, consisting of 1.5 μg total DNA and varying ratios of SecR-GFP2 to SecR-YFP plasmids (1:1, 1:2, 1:3, and 1:5), was delivered using Lipofectamine 3000 (Invitrogen) according to the manufacturer’s protocol. Following a 24-hour incubation post-transfection, the cell samples were transported to the imaging system in a portable incubator. Prior to imaging, the DMEM based cell media was replaced by Dulbecco’s Phosphate Buffered Saline (DPBS). All micro-spectroscopic scans of the cells were obtained at room temperature.

#### Spectral unmixing of fluorescence image and determination of pixel-level apparent FRET efficiencies ($E_{app}$) values and concentrations

The composite emission spectrum of each pixel was unmixed into donor and acceptor components using a General Least-Squares (GLS) method [55–57]. Elementary spectra for the donor and acceptor were derived from fluorescence images of cells expressing only SecR-GFP2 (donor) or only SecR-YFP (acceptor). The unmixed data produced 2D spatial distribution maps of the donor signal in the presence of the acceptor ($k^{DA}$) and acceptor signal in the presence of donor ($k^{AD}$). The $k^{DA}$ and $k^{AD}$ values were multiplied with the area underneath their respective elementary spectrum ($w^{D}$ and $w^{A}$, respectively) to obtain the donor fluorescence emission in the presence of the acceptor, $F_{exc\text{\#}}^{DA}=k_{exc\text{\#}}^{DA}\cdot w^{D}$, and acceptor emission in the presence of the donor, $F_{exc\text{\#}}^{AD}=k_{exc\text{\#}}^{AD}\cdot w^{A}$ [21]. The subscript exc# refers to which of the excitation wavelengths (*1* or *2*) the dataset was collected from.

The apparent FRET efficiency ($E_{app}$) represents the ratio between the donor fluorescence lost due to FRET ($F^{D,FRET}$) and the fluorescence of the donor in the absence of acceptor ($F^{D}$) within a mixture of oligomers of different sizes and abundances:

(1) $$E_{app}=\frac{F^{D,FRET}}{F^{D}}.$$

At one of the excitation wavelengths used in this study (800 nm) direct excitation of the acceptor was minimal, simplifying Equation (1) to [21, 29]:

(2) $$E_{app}={\left[1+\frac{Q^{A}}{Q^{D}}\frac{F_{2}^{DA}}{F_{2}^{AD}}\right]}^{-1},$$

where $Q^{D}=0.55$ is the quantum yield of the donor [58] and $Q^{D}=0.61$ is the quantum yield of the acceptor [59]. Subscript *2* indicates that fluorescence emission was obtained from the excitation of the sample using the *2*^*nd*^ excitation wavelength (800 nm). The donor and acceptor concentration values were determined in each pixel from the measured $F_{exc\text{\#}}^{DA}$ and $F_{exc\text{\#}}^{AD}$ values following the protocol established by Paprocki et al [31], which is summarized in Supplementary Methods Section SM4.

#### Assembly of $E_{app}$ histograms and FRET spectrogram

$E_{app}$ was calculated for each pixel in a micro-spectroscopic image using Equation (2). $E_{app}$ values from pixels where both donor and acceptor intensities exceeded the standard deviation of the noise in said pixel were selected and organized into a number of different histograms. Each histogram was generated from the $E_{app}$ values from a small group of pixels (<100 pixels), or segments, taken from regions corresponding to the cell membrane, as illustrated in Figure 2. The membrane segments in each image were generated by a computer program, detailed in Supplementary Method Sections SM5 and SM6. The two most prominent peaks of each histogram were selected using the peak selection algorithm outlined in Supplementary Methods Section SM7. A separate protomer concentration value was calculated for each peak position, estimated as the median of pixel-level receptor concentrations from all pixels included in the formation of the extracted peak. A FRET spectrogram, also dubbed “FRET spectrogram,” was constructed using $E_{app}$ peak values whose concentration values fell between 0 to 48 receptors per pixel.

The FRET spectrogram thus assembled (Supplementary Figure 1) has a distribution of distinct peaks, each of which representing a frequently occurring $E_{app}$ value characterizing a specific pathway for energy transfer between donors and acceptors within hSecR oligomers, such as dimers and tetramers, or within mixtures of different oligomeric sizes (see below and Ref. [57]). We observed that altering the lower bound of the first FRET spectrogram bin while keeping the bin width constant changed the appearance of the FRET spectrograms, with significant differences occurring in both the positions and amplitudes of the peaks, as shown in Supplementary Figure 1. We maximized the information content in the FRET spectrogram (i.e., the visibility) by choosing a bin origin that resulted in the maximum value of the peak visibility defined by

(3) $$Peak\;visibility=\sum \frac{1}{2}(Right\;amplitude+Left\;amplitude),$$

where ‘Right amplitude’ and ‘Left amplitude’ represent the peak height above the nearest minimum on the right and left, respectively. The sum runs over all detected peaks identified using the peak selection algorithm. The FRET spectrogram with the highest peak visibility was chosen for further analysis.

The image analysis process up to and including the FRET spectrogram generation, was performed using a dedicated software for FRET spectrometry analysis, named OptiMiS DC, which can be downloaded from https://github.com/Raicu-Lab-UWM/OptiMiS-DC. A compiled version of the software can be downloaded from https://sites.uwm.edu/raicu-research-group/software.

### FRET spectrogram analysis using molecular dynamics (MD) simulations

#### Construction of SecR-GFP2 structure files

GFP2 [58] was based on a green fluorescent protein structure obtained from the protein data bank (PDB code: 1EMC [60]) and constructed by modifying various amino acids. The active SecR structure (PDB code: 6WZG [1]) was also obtained from the protein data bank and modified by removing the ligand, the G-protein complex, and adding residues at the C-terminus. The modified structure underwent a 9 μs MD simulation (See Supplementary Figure 3). GFP2 was then attached to the C-terminus of the inactive (i.e., MD simulated) SecR. See Supplementary Methods SM8 for detailed amino acid modifications.

SecR-GFP2 dimer structures were built by aligning two SecR-GFP2 copies in Chimera, rotating each monomer about the axis perpendicular to the membrane plane until TM domains of interest (referred to as the “dimer interface”) faced opposite directions, and moving the monomers apart until the van der Waals surfaces of the closest contact points between the dimers did not significantly overlap. One uniquely crafted MD-starting structure featuring the TM4 interface, termed TM4/TM4 hydrophobic, was created by orienting two SecR-GFP2 protomers such that the TM4 domains close-proximity residues matched those from previous studies [16, 17]. For all other starting dimer structures, no preference was given as to which residues started in closest contact.

Tetramers were assembled by bringing two SecR-GFP2 dimer copies together, ensuring that the contact interface, known as the “tetramer interface,” involved different transmembrane domains than those at the “dimer interface”. A total of eight MD-starting tetrameric structures (listed in Supplementary Table 3) were constructed and simulated. All structures were produced using the Chimera software [61].

#### MD simulations protocol

All MD simulations were conducted using the GROMACS software package [38] and implemented on the Mortimer Faculty Research Cluster located at UW-Milwaukee. The PDB files of the oligomer structures (both dimers and tetramers) were first converted from all atomic (AA) representation to a coarse grained (CG) model using martinize.py (ver 2.4) [39]. The CG model incorporated an elastic network (ElNeDyn) using the -ff elnedyn22 option in the “martinize” command. The CG oligomer structure was embedded in a CG membrane, consisting of 90% DPPC and 10% cholesterol using the Martini script insane.py [62]; the membrane thickness was set to be 2.5 nm. The dimensions of the simulation box were 24 nm × 24 nm × 20 nm, with periodic boundary conditions used.

The system was energy-minimized using a steepest descent algorithm, terminating when the maximum force reached less than 1000 kJ mol^−1^ nm^−1^. Subsequently, eight 1 μs simulations were conducted for each tetramer orientation, all starting from the same minimized membrane/protein system. For each run, an initial equilibration phase, lasting 10 ns with time steps of 20 fs, was executed. Temperature control (293 K) was achieved through the velocity-rescale thermostat (coupling constant: 1 ps), and pressure was maintained at 1 bar by the Berendsen barostat (coupling constant: 12 ps). Subsequently, simulation runs were performed, with temperature coupling mirroring the equilibration, and the Parrinello-Rahman barostat (coupling constant: 12 ps) used for pressure control. Electrostatic interactions were computed using the reaction field method, with a real space cutoff of 1.1 nm, and the Van der Waals interaction cutoff was set at 1.1 nm. The simulation run was performed for 1 μs with a time step of 20 fs and the coordinates of the system were saved every 100 ps.

#### FRET efficiency calculation of oligomeric complexes from MD simulation coordinates

The FRET efficiency ($E_{n,k,q,f}^{MD}$ ) of an oligomeric configuration, q, comprising n protomers and k donors was determined from the distance $r_{i,j,q,f}$ and orientation factor $\kappa_{i,q,f}^{2}$ between all possible donor-acceptor pairs within the oligomeric complex for a given coordinate frame, f, using the following relation:

(4) $$E_{n,k,q,f}^{MD}=\frac{1}{k}\sum_{i=1}^{k}\frac{\sum_{j=1}^{n-k}\;\;3\kappa_{i,j,q,f}^{2}{\left(\frac{R_{0}}{r_{i,j,q,f}}\right)}^{6}}{2+\;\sum_{j=1}^{n-k}3\kappa_{i,j,q,f}^{2}{\left(\frac{R_{0}}{r_{i,j,q,f}}\right)}^{6}},$$

where $R_{0}=5.64\;nm$ is the Forster distance for the GFP2-YFP pair [58]. The sums over i and j are over all Ds and As, respectively, in the configuration q. Details regarding the extraction of $r_{i,j,q,f}$ and $\kappa_{i,j,q,f}^{2}$ for all possible donor-acceptor pairs in each frame are given in Supplementary Methods Section SM9.

A calculation of $E_{4,k,q,f}^{MD}$ was performed separately for each of 14 possible tetramer configurations (i.e., q values obtained by permutating the placement of donors and acceptors) at every frame throughout the simulation. For a given q value, the frame-level FRET values were averaged over the frames spanning 100 ns to 1 μs across eight independent simulations. This process yielded an average FRET value, $E_{4,k,q}^{MD}$, corresponding to each q value. The same distance, $r_{a,b}$, and orientation factor, $\kappa_{i,j}^{2}$, data was used to compute the 14 possible apparent FRET efficiency values observed for different q values.

To calculate the FRET efficiency within these trimeric configurations, we applied Equation (14) to every frame of the tetramer MD simulations. When summing over protomers in Equation (14), we excluded the dissociated protomer to accurately represent the trimeric state. The resulting FRET efficiencies for the trimer configurations are denoted as $E_{3,k,q}^{MD}$ and $E_{3,k,q^{*}}^{MD}$.

FRET efficiencies for all dimer orientations were determined using Equation (14) applied to separate simulations, each involving only two protomers. The resulting FRET efficiencies for the dimer configurations are denoted as as $E_{2,k,q}^{MD}$ and $E_{2,k,q^{*}}^{MD}$. The dimer FRET efficiencies were calculated using the same number of frames and total simulations as those used for the tetramers and trimers.

#### Analysis of experimental FRET spectrograms using MD simulated data

To determine the FRET efficiency for a specific oligomeric configuration, q, we calculated the weighted average across all dissociation states based on the relative time spent in each state. This weighting considers the probability of dissociation for the TM domains forming TMA, denoted as $P_{A}$, and the probability of dissociation of the TMB domains, $P_{B}$. The overall FRET efficiency, incorporating all sixteen dissociation states, φ, is calculated as follows:

(5) $$E_{mix,k,q}^{MD}=1-\frac{1}{k}\sum_{m=1}^{m=16}\;P_{m}\left[k_{m,q}\left(1-E_{m,q}\right)+\left(k-k_{m,q}\right)\right].$$

where $P_{m}$ is the probability of being in dissociation state m, determined by the parameters $P_{A}$ and $P_{B}$ . The sum over 16 dissociation states reflects the inclusion of two distinct types of “conjugate” tetramers, which are associated with the same pair of interacting TM domains. The term $E_{m,q}$ represents the FRET efficiency of the remaining intact portion of the oligomer which did not undergo dissociation, i.e., the $E_{n,k,q}^{MD}$ values obtained from MD simulations. Additionally, $k_{\varphi ,q}$ represents the number of donors still participating in an interaction, excluding free monomeric donors that have dissociated from the original tetramer.

Using the values of the MD-simulated FRET efficiencies, the oligomer dissociation model was then employed to fit the experimental FRET spectrogram. The theoretical fitting function employed to model the FRET spectrograms was a composite of 14 Gaussians, each corresponding to one tetramer configuration. The fitting function takes the form:

(6) $$S\left(E_{app}\right)=\sum_{q}\;A_{q}exp\left[-\frac{{\left(E_{app}-E_{mix,k,q}^{MD}\right)}^{2}}{2\sigma_{q}^{2}}\right]$$

where $A_{q}$ is the amplitude of the *q*^th^ Gaussian and $\sigma_{q}$ its standard deviation. The mean values of the 14 Gaussian functions comprising the oligomer dissociation model were completely determined by the fitting parameters $P_{A}$ and $P_{B}$ along with the MD simulated FRET efficiency values, $E_{n,k,q}^{MD}$, according to Equation (5). $S(E_{app})$ was fitted to experimental $E_{app}$ FRET spectrograms by adjusting the values of $A_{q}$ and $\sigma_{q}$ along with the parameters $P_{A}$ and $P_{B}$ to minimize the fitting residual,

(7) $$Res=\sqrt{\sum_{i}\;(Data\;-\;Fit{)}^{2}}$$

where ‘Data’ stands for the experimentally determined counts in the FRET spectrogram and ‘Fit’ for the simulated values given by Equation (6).

**Figure 5.**
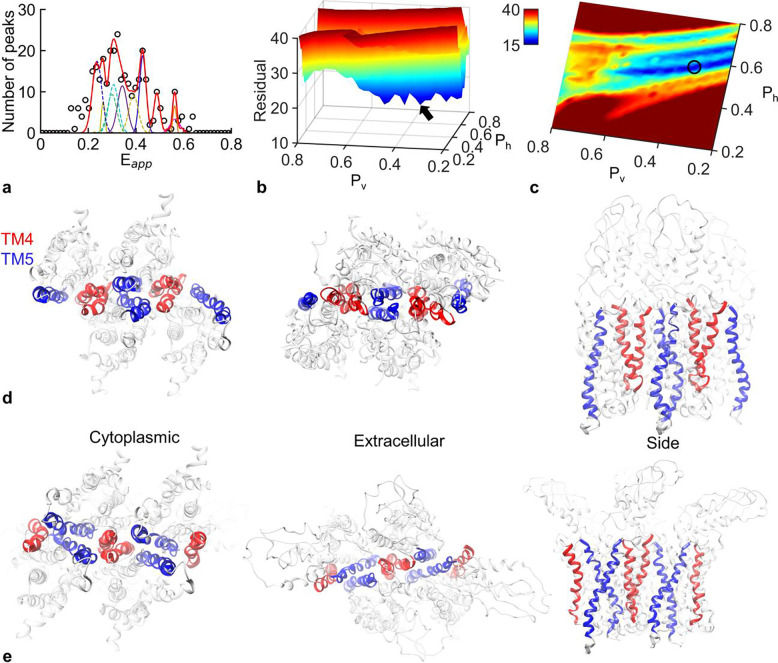
The quaternary structure of hSecR. **(a) $E_{app}$** FRET spectrogram (empty circles) for relatively low concentrations of protomers (0–48 protomers/pixel) obtained from HEK-293 cells co-expressing hSecR-GFP2 and hSecR-YFP. The FRET spectrogram was fitted (solid red line) using the oligomer dissociation model (see Equation 5) along with the MD simulated FRET efficiency values from a number of different TM domain conformation pairs (see Figure 3 and Supplementary Table 3). The best overall fit was obtained using the TM domain conformation pair of TM4 (hydrophobic) and TM5. **(b)** A surface plot illustrates the fitting residual (represented by both height and color) for different dissociation probabilities, $P_{A}$ and $P_{B}$. These probabilities were obtained from fitting the $E_{app}$ FRET spectrogram using FRET efficiency values from MD simulations of tetramers that began with interactions at the TM4 (hydrophobic) and TM5 interfaces. A black-colored arrow is directed towards the lowest value of the residual on the surface plot. **(c)** top view of the surface plot in (b). A black circle is centered at the location of the lowest residual value on the plot**. (d)** Ribbon representations from various perspectives show the two conjugate tetramers that make up the TM domain conformation pairing which resulted in the best fit for the hSecR FRET spectrogram. The average dissociation probabilities were: $P_{A}=\;0.56\;\pm \;0.06$ (TM4/TM4) and $P_{B}\;=\;0.39\;\pm \;0.15$ (TM5/TM5). These values were used to calculate the average fraction of oligomers of different sizes. The results were: monomers, 0.61 ± 0.03; dimers, 0.22 ± 0.07; trimers, 0.12 ± 0.04; tetramers, 0.05 ± 0.01.

**Figure 6.**
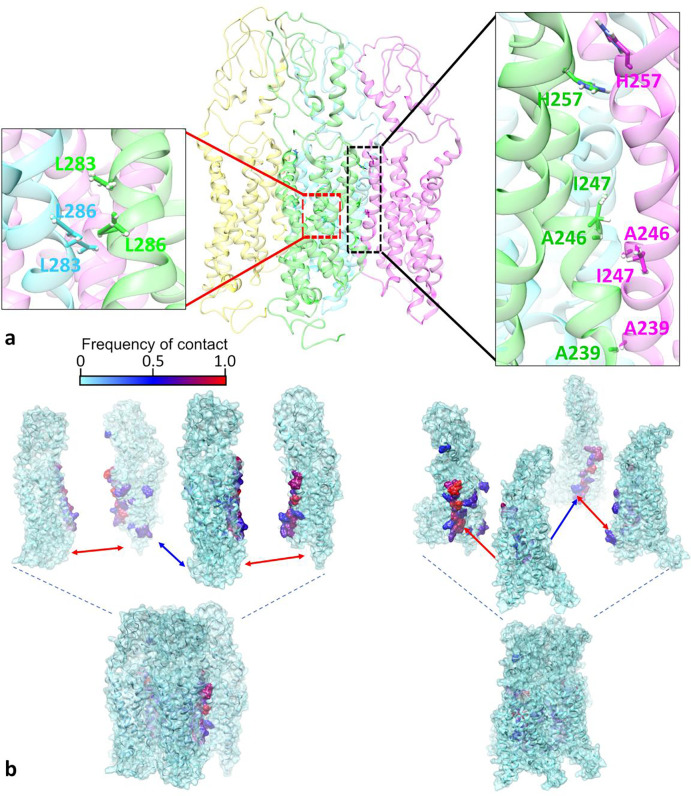
Binding interface details of the hSecR oligomeric structure featuring (a) ribbon and (b) three-dimensional surface shading representations of the tetramer. The side-view ribbon representation in (a) shows the residues with the highest contact frequency in the TM4/TM4 (hydrophobic):TM5/TM5 tetramer structure after a 1 μs-long MD simulation. Protomers are highlighted in distinct colors. The insets provide magnified views of the two interfaces, with the top-contact residues shown in stick representation (oxygen, red; nitrogen, blue; hydrogen, white). The TM4/TM4 binding interface includes the residue pairs HIS257/HIS257, ALA246/ILE247, ALA239/ALA239. The TM5 interaction interface includes the residue pairs LEU283/LEU286. The three-dimensional surface representation in (b) shows the hSecR tetramer (bottom row) with “exploded” view perspectives (top) presented parallel to a plane bisecting the two protomers interacting at the TM5 interface (left panel) and orthogonal to this plane (right panel). The TM domains in each hSecR protomer are displayed as cyan ribbons with overlaid surfaces. Residue-specific coloration on the surface corresponds to the residue-level contact frequency, visually representing intermolecular interactions within the tetramer. The red arrows point to interfaces formed by TM4 domains and the blue arrows indicate interfaces formed by TM5 domains.

**Figure 7.**
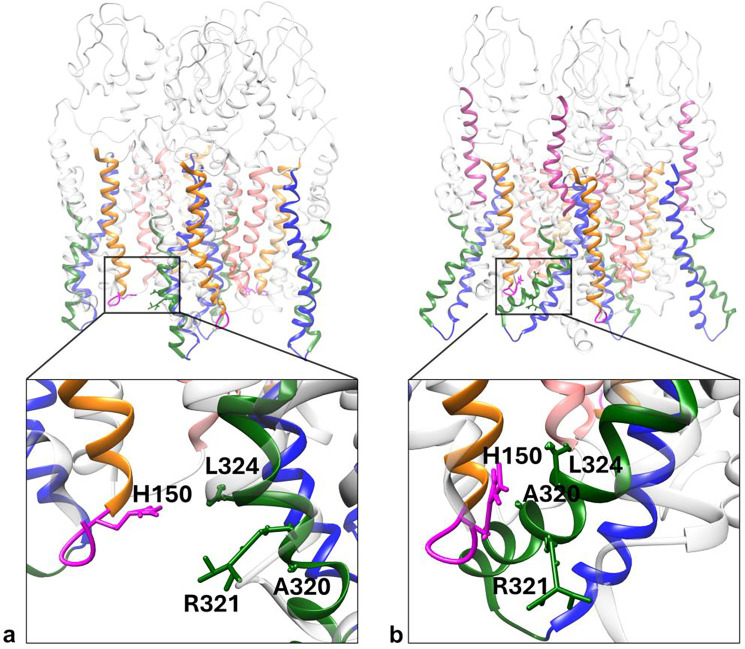
Illustration of a potential tetramer-stabilizing binding interface created through the outward movement of the cytoplasmic sized of TM6 upon activation of hSecR with secretin ligand. The TM4/TM4 (hydrophobic) and TM5/TM5 hSecR tetramer assembled from **(a)** inactive protomers and **(b)** ligand-bound activated protomers and simulated with a short equilibration run. In the liganded state, the cytosolic part of TM6 points outward toward the intracellular loop 1 (ICL1) of a neighboring protomer. Insets show the magnified regions within the black rectangle where TM6 comes near ICL1 in the active structure; the residues in closest contact, determined from average distances computed over several equilibrated runs are labeled. These interactions appear crucial in supporting the stability of the TM4/TM4-mediated interface. Color coding: red, TM4; blue, TM5; green, TM6; pink, ICL1; violet, secretin ligand.

To ensure that we obtained the set of fitting parameters that resulted in a global minimum of the fitting residual, we employed a fitting procedure where the values of $P_{A}$ and $P_{B}$ were restricted to a narrow fitting range of 0.02. The lower and upper bounds of this narrow range were systematically iterated over their physically meaningful range (i.e., from 0 to 1). At each step, we fine-tuned the amplitudes and standard deviations of the Gaussians that constitute the oligomer dissociation model until the fitting residual was minimized. The spectrogram fitting which resulted in the lowest overall fitting residual is displayed in Figure 5a. A two-dimensional map illustrating the fitting residual as a function of different $P_{A}$ and $P_{B}$ values is presented in Figure 5b and 5c. The combination of $P_{A}$ and $P_{B}$ values that resulted in the lowest residual across this 2D residual map was considered the best fit for a TM domain conformation pair.

#### Identification of contact residues and contact domains

Simulated tetramer structures were further analyzed to determine which residues and TM domains were most frequently in contact at the binding interfaces throughout each simulation. MD simulation frames separated by 10 ns intervals, from 0.1 μs to the conclusion of the 1-μs MD trajectory, were examined. Residues were considered ‘in contact’ when their separation was less than 5 Å. The contact frequency between two domains represents the percentage of frames in which those domains were in contact relative to the total number of frames. Similarly, individual transmembrane (TM) domains were considered ‘in contact’ if there was a pair of atoms – one from each facing residue or domain in opposing protomers – with a separation less than 5 Å. The identification of these contacts was conducted using the ‘findclash/contact’ command in Chimera. The average frequency of contacts was determined across eight independent simulations of the tetramer structure.
